# How far do tadpoles travel in the rainforest? Parent-assisted dispersal in poison frogs

**DOI:** 10.1007/s10682-019-09994-z

**Published:** 2019-07-05

**Authors:** Andrius Pašukonis, Matthias-Claudio Loretto, Bibiana Rojas

**Affiliations:** 1 Department of Biology, Stanford University, 371 Serra Mall, Stanford, CA 94305 USA; 2 Department of Cognitive Biology, University of Vienna, Althanstrasse 14, 1090 Vienna, Austria; 3 Department of Biological and Environmental Sciences, University of Jyväskylä, PO Box 35, 40014 Jyväskylä, Finland

**Keywords:** Informed dispersal, Parental care, Tadpole transport, Resource use, Dendrobatidae

## Abstract

Parents can influence offspring dispersal through breeding site selection, competition, or by directly moving their offspring during parental care. Many animals move their young, but the potential role of this behavior in dispersal has rarely been investigated. Neotropical poison frogs (Dendrobatidae) are well known for shuttling their tadpoles from land to water, but the associated movements have rarely been quantified and the potential function of tadpole transport in dispersal has not been addressed. We used miniature radio-transmitters to track the movements of two poison frog species during tadpole transport, and surveyed pool availability in the study area. We found that parental males move farther than expected by the distance to the nearest pool and spread their offspring across multiple pools. We argue that these movement patterns cannot be fully explained by pool quality and availability, and suggest that adaptive benefits related to offspring dispersal also shape the spatial behavior of parental frogs.

**Electronic supplementary material:**

The online version of this article (10.1007/s10682-019-09994-z) contains supplementary material, which is available to authorized users.

## Introduction

The local physical and social environment can have a strong influence on animal dispersal (i.e., context-dependent dispersal, Bowler and Benton 2005; Matthysen 2012). Dispersing individuals may integrate the environmental factors experienced at the present and learned in the past (informed dispersal *sensu* Clobert et al. 2009), resulting in complex movement strategies. Adults are usually more experienced than their offspring, but dispersal is more common in early life stages (Clobert et al. 2012). Parents, however, can influence subsequent offspring dispersal by evaluating and choosing breeding sites and by directly moving offspring during parental care (Bonte et al. 2007; Matthysen et al. 2010; Clobert et al. 2012). For example, female wolf spiders show greater mobility while carrying spiderlings, thus influencing offspring natal dispersal patterns and potentially reducing kin competition (Bonte et al. 2007). Animals as diverse as arachnids and mammals carry their young to protect them during their most vulnerable stage (e.g., Ross 2001; Wolff et al. 2015). Offspring transport may also promote adaptive movement strategies that favor offspring dispersal. While factors such as habitat selection, inbreeding avoidance, and kin-competition are at the core of dispersal theory, the role of parental mobility in offspring dispersal has received little attention so far.

**Fig. 1:**
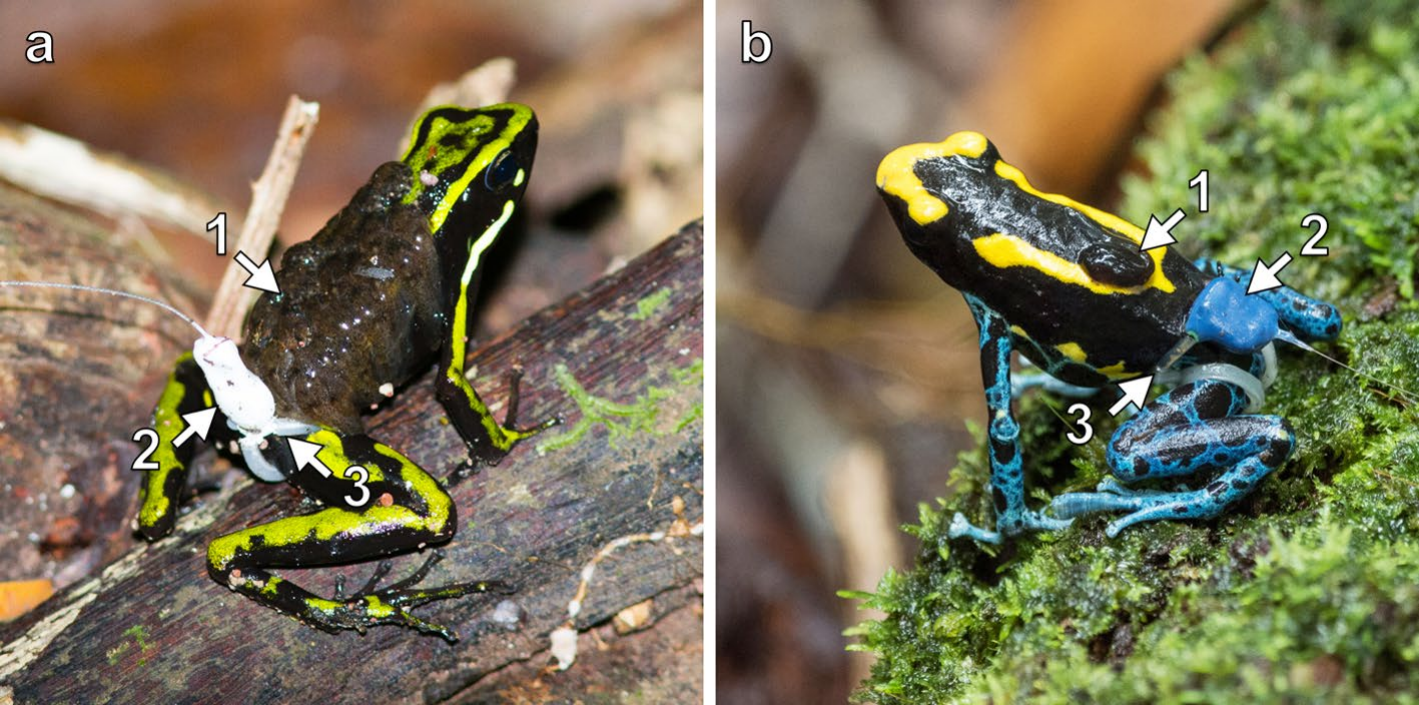
Photographs of the two study species: **a***Ameerega trivittata* and **b***Dendrobates tinctorius* transporting tadpoles while wearing a radio-transmitter. *Ameerega trivittata* typically transports 15–30 tadpoles while *D. tinctorius* only transport one or two tadpoles. The numbers and arrows indicate: (1) tadpoles, (2) radio-transmitter, and (3) a silicone waistband for attachment. (Color figure online)

The transport of eggs, tadpoles, and froglets is widespread in anuran amphibians, especially in Neotropical poison frogs (Dendrobatidae) (Wells 2007). Poison frogs are terrestrial and lay their eggs on land, a common strategy among tropical amphibians, presumably shaped by aquatic predator avoidance (Magnusson and Hero 1991; Duellman and Trueb 1994). However, most poison frog tadpoles are aquatic and thus need to be taken by one of their parents from land to water (Fig. 1). Depending on the species, tadpoles are carried by males or females, singly or in groups, and released in terrestrial or arboreal pools ranging from large streams to small water-filled plants (reviewed in Summers and McKeon 2004; Wells 2007). This diversity of parental behaviors has been primarily viewed as a result of trade-offs between water volume-dependent food availability and predation risk inside the pools (Weygoldt 1987; Summers and McKeon 2004; Brown et al. 2010; Summers and Tumulty 2013). Tadpole transport allows parents to make flexible decisions when choosing the best microhabitat for their offspring (Summers and McKeon 2004; Brown et al. 2009; Ringler et al. 2018). In addition, it allows frogs to disperse their offspring over large areas and distribute them among multiple sites (Erich et al. 2015; Beck et al. 2017). Similar to other forms of dispersal (for a review see Bowler and Benton 2005), the benefits of tadpole transport may also include the colonization of new areas, reduced kin competition and inbreeding, and the spread of risks between multiple resources. However, the role of tadpole transport in dispersal has been rarely addressed. In this study, we aim to highlight that offspring dispersal could play an important role in shaping parental spatial behavior in poison frogs, and possibly in other taxa that transport their young.

We used miniature radio-transmitters to track parental frog movements during tadpole transport in two poison frog species with male parental care, but otherwise contrasting reproductive strategies. We were particularly interested in establishing whether the frogs use the nearest pool available. Tadpole transport requires energy and time, and may increase exposure to predators and reduce mating opportunities (Beck et al. 2017; Ringler et al. 2013; Wells 2007; this study). Therefore, males should try to minimize the distance and duration of tadpole transport unless there are direct benefits of traveling farther. However, if the benefits related to active offspring dispersal have shaped the spatial behavior of poison frog parents, we would expect more complex movement patterns to emerge.

## Methods

### Study species and sites

We radio-tracked tadpole transporting males of two poison frog species, *Ameerega trivittata* (Three-striped poison frog, abbreviated as At for methods and results) and *Dendrobates tinctorius* (Dyeing poison frog, abbreviated as Dt for methods and results). Both species are diurnal, breed throughout the rainy season, and are locally common but allopatric throughout most of their range (AmphibiaWeb 2019). In both species, males transport tadpoles from home territories to water and return back to them after tadpole transport (Silverstone 1975, 1976; Roithmair 1994a, b; Rojas 2014, 2015; Rojas and Pašukonis 2019; this study). The two species differ significantly in other aspects of their reproductive behavior. *Ameerega trivittata* males call and defend small territories where mating takes place (Roithmair 1994a, b). Clutches of ~ 40 eggs are laid in the leaf-litter where they develop for 15–22 days before the male transports tadpoles simultaneously to small terrestrial pools and streams (Acioli and Neckel-Oliveira 2014; Roithmair 1994a, b; this study Fig. S1a–c). Tadpoles are omnivorous and several hundred tadpoles can be found in a single pool (Luiz et al. 2015). In captivity, tadpoles metamorphose after 40–90 days and frogs reach maturity within 1 year (Lötters et al. 2007). *Dendrobates tinctorius* males show aggressive behavior but lack loud advertisement calls and do not always defend exclusive areas (Born et al. 2010; Rojas and Pašukonis 2019). In our study area, pairs lay small clutches of 2–5 eggs. After ~ 15 days of development, males shuttle 1 or 2 tadpoles simultaneously to small pools formed in palm bracts or tree-holes at variable heights (Rojas 2014, 2015; this study Fig. S1d–f). Tadpoles are primarily carnivorous and cannibalistic, and typically less than 10 tadpoles are found in one pool (Rojas 2014, 2015). In the field, tadpoles metamorphose after approximately 2 months (B. Rojas, pers. obs.) and take up to 18 months to mature in captivity (Lötters et al. 2007).

**Fig. 2:**
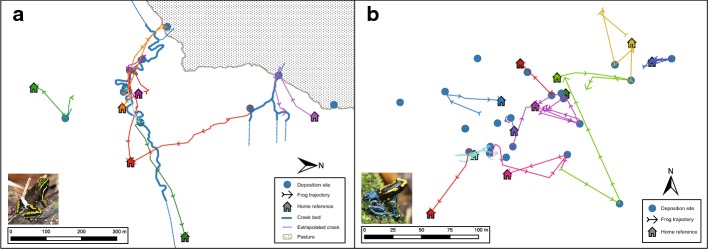
Map of the study area showing the movements during the tadpole transport of **a** seven *A. trivittata* males and **b** 11 *D. tinctorius* males. Blue circles represent confirmed tadpole deposition sites; house symbols represent approximated start location of the tadpole transport; each line corresponds to a transport event and each color represents a different individual. **a** Blue solid and dashed lines mark creek beds, which provided most deposition sites; dotted area corresponds to the forest edge. The shown trajectories do not represent complete movement patterns because some frogs were first detected already outside their home areas and near the deposition sites. Note the difference in map scales between the two species. (Color figure online)

Data for At were collected around the onset of the rainy season in October and November 2014, at the Panguana Biological Field Station inside “Área de Conservación Privada Panguana” on the lower Río Llullapichis, Amazonian Peru (9°35′S, 74°48′W). We monitored an area of approximately 30 ha of rainforest bordering a pastureland on one side. We mapped all terrestrial water bodies found during our study with a GPS/GIS device (MobileMapper 10; Ashtech/Spectra Precision) and ArcPAD 10 (ESRI) software. Some stream sections were not accessible and were mapped by extrapolating mapped parts of the stream. Water bodies were found opportunistically, mostly when tracking tadpole-transporting frogs. Most water bodies consisted of series of partially separated pools in the stream bed that were intermittently connected by flowing water after heavy rainfalls (Fig. 2). Because of the proximity and connectivity of individual pools, the entire stream beds were considered as a tadpole deposition sites. We observed frogs using all mapped water bodies, except for one larger permanent pond, which was excluded from the analysis.

Data for Dt were collected during the mid-rainy season in February–March 2016 and 2017 near the Camp Pararé field site at the CNRS Nouragues Ecological Research Station in the Nature Reserve Les Nouragues, French Guiana (4°02′N, 52°41′W). We monitored an area of approximately 4 ha of terra-firme rainforest. We mapped all visible water bodies and trees that were climbed by tadpole-transporting frogs. All pools visited by frogs that were accessible for inspection contained tadpoles of Dt and were considered as suitable tadpole deposition sites. In addition, we considered all trees that were climbed by tadpole-transporting frogs as potential deposition sites. *Dendrobates tinctorius* plot was sampled more evenly than At plot because of the smaller study area, more open understory, and longer study period. However, because Dt primarily use small pools above the ground, the pools were harder to detect and most deposition sites were detected only by tracking tadpole-transporting frogs.

### Tagging and tracking

*Ameerega trivittata* males (n = 9) were all captured during tadpole transport. One frog was recaptured on two consecutive tadpole transport events. *Dendrobates tinctorius* males were captured either during tadpole transport (n = 5) or were already tagged before tadpole transport (n = 6) as part of another study. From these 11 Dt males, two frogs were observed on two and three consecutive transport events. One individual was tracked on two different years. Frogs were kept inside a plastic bag or a net-cage for 15 to 105 min for the preparation and fitting of the transmitter, but handled only for a few minutes at a time. Frogs were equipped with miniature radio transmitters (BD2X, Holohil Systems Ltd.; NTQ2, Lotek Wireless Inc.; PicoPip, Biotrack Ldt; V5, Telemetrie-Service Dessau) attached externally using a waistband or a harness made of silicone materials (Fig. 1; Supplementary video). The tags constituted approximately 10% of the total frog weight (Dt: frog weight 3.5–4.2 g, tag weight 0.35–0.4 g; At: frog weight 3.8–5.6 g, tag weight 0.36–0.5 g). After release, the frogs were located 2 to 12 times a day during the daylight hours using a portable radio-tracking receiver (Sika, Biotrack Ltd.) and a flexible Yagi-antenna (Biotrack Ltd.).

We considered a tadpole transport event to be over after a frog deposited all tadpoles and returned back to their presumed home territory after which the frog showed no directional movement for at least 24 h. One At was predated by a snake after tadpole deposition (Fig. S2). In two cases tracking was terminated due to skin injuries from tag attachment. The mapping was done using a combination of GPS and local references established with traditional survey methods (see Ringler et al. 2016). The estimated relative GPS error was approximately 5–8 m. For smaller scale movements we measured the distance and the direction from the previous location using a compass and a laser distance-meter. All data were recorded using a handheld GPS/GIS device.

### Data analysis

Data analysis and visualization were done in QGIS v2.14 (https://www.qgis.org/) and R statistical software (http://www.R-project.org/). For each tadpole transport event, we calculated (1) total duration, (2) cumulative path length, (3) straight-line distance from home to the farthest tadpole deposition site used (i.e., observed pool distance), and (4) straight-line distance to the nearest identified pool site (i.e., nearest pool distance). We did not quantify the home ranges or territories in this study, but approximated the origin of the tadpole transport by a single location termed “home reference” within the presumed home territory. Because most frogs were first located on the way to or at the pools, we used the last point of their homing trajectory as their home reference (Fig. S3). When the exact starting location of the tadpole transport was known or when homing tracking was terminated prematurely due to predation or injury, we used the first point of the trajectory as the home reference. The distances to pools for each frog were calculated from their respective home references. To estimate the cumulative path travelled for incomplete trajectories, we added the straight-line distance from the last point of the trajectory (i.e., home reference) to the location of the first observation. Because of the incomplete tadpole transport trajectories, the total duration and the distances of the transport are very conservative and likely underestimated. We performed a Mann–Whitney–Wilcoxon Test to compare the total durations, observed pool distance, and cumulative distances between the two species, and a paired Wilcoxon Signed-Rank Test to compare the observed and nearest pool distance for each species.

## Results

We successfully tracked eight At and 15 Dt tadpole transport events (seven and 11 different individuals, respectively) (Fig. 2). *Ameerega trivittata* males transported 15 to 32 tadpoles (mean ± SD = 22.1 ± 6.2) and deposited them in standing water pools in a partially dried-up stream bed (n = 6) or flooded meadow on the edge of the forest (n = 2). Frogs visited and deposited tadpoles in one or two separate water bodies within a single transport event. *Dendrobates tinctorius* transported 1 to 2 tadpoles and deposited them in water-filled tree holes at 1–10 m above ground (n = 5), presumed tree holes above 10 m and thus out of sight (n = 6), standing water inside fallen trunks (n = 3), and palm bracts (n = 1). *Dendrobates tinctorius* visited up to three deposition sites within one transport event. When transporting two tadpoles (n = 4) frogs deposited both tadpoles in the same pool. When the same Dt male was observed during more than one transport event (n = 3) frogs deposited tadpoles in different sites during each event.

*Ameerega trivittata* used tadpole deposition sites farther from home areas than Dt (mean observed distance_AT_ ± SD = 215 ± 109 m, range 96–371 m; mean observed distance_DT_ ± SD = 39 ± 29 m, range 6–121 m; Wilcoxon test: W = 118, *p* < 0.001); traveled longer cumulative paths (mean path_AT_ ± SD = 486 ± 194 m, range 215–766 m; mean path_DT_ ± SD = 109 ± 63 m, range 17–260 m; Wilcoxon test: W = 119, *p* < 0.001); and spent longer time away from the home area (mean duration_AT_ ± SD = 79.6 ± 41.5 h, range 5.9–140 h; mean duration_DT_ ± SD = 14.9 h ± 10.9 h, range 2.5–33.8 h; Wilcoxon test: W = 108, *p* = 0.001).

**Fig. 3:**
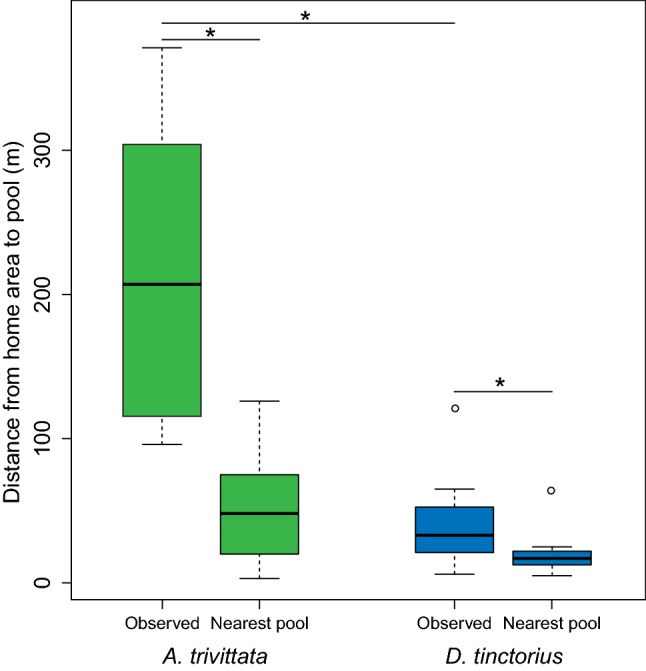
Boxplot illustrating the difference in distance between the observed tadpole transport distances and the nearest known pool available for each tracked frog and species. Asterisks denote statistically significant differences based on Mann-Whitney-Wilcoxon and Wilcoxon Signed-Rank Tests (*p* < 0.05). (Color figure online)

Males of both species traveled significantly longer distances than the distance to the nearest available pool (mean pool distance_AT_ ± SD = 52 ± 41 m, range 3–126 m; Wilcoxon paired test: V_AT_ = 28, *p* = 0.02; mean pool distance_DT_ ± SD = 19 ± 14 m, range 5–64 m; Wilcoxon paired test: V_DT_ = 55, *p* = 0.006; Fig. 3).

## Discussion

Males of both species carried their offspring to several sites farther away than the closest pool available from their respective home areas. The frogs moved directly toward distant pools and sometimes ignored nearby pools that had been used by other individuals even when passing next to them. We thus suggest that patterns of pool availability and quality cannot fully explain the observed movement patterns. We propose that adaptive benefits related to offspring dispersal could also shape the spatial behavior of parental poison frogs.

Resource quality plays an important role in poison frog pool choice (e.g., Brown et al. 2008; McKeon and Summers 2013; Schulte et al. 2013; Ringler et al. 2018), but the assessment of pool quality alone is unlikely to fully account for the large movement extent observed in our study. In most cases, multiple pools were available closer to the home territory, but the frogs did not approach them before moving to more distant pools. Also, frogs ignored some pools that were used by other conspecifics coming from farther away, even when passing in their immediate vicinity. On several occasions, we observed *A. trivittata* males leaving the forest to deposit tadpoles in a flooded pasture (Fig. 2a), an unusual habitat for a forest species. Dispersal is a key component for understanding the spatial behavior of temperate-region pond-breeding amphibians (Cayuela et al. 2018; Pittman et al. 2014; Sinsch 2014); we propose that parental dispersal in poison frogs might convey adaptive benefits that are important to consider for a better understanding of their behavior.

Dispersing offspring farther than the nearest available pool, and using multiple pools, may entail numerous benefits. Chief among them are reduced competition and inbreeding risk between parents and offspring, and spreading the risk of tadpole predation and pool desiccation, both of which are high in rainforest pools (Magnusson and Hero 1991; Richter-Boix et al. 2011). The use of multiple pools has been reported in other poison frogs (Summers 1990; Brust 1993; Poelman and Dicke 2007; Brown et al. 2008), and experimental studies using artificial pools revealed that males of the poison frog *Allobates femoralis* remember and use multiple pools over large areas (Erich et al. 2015; Beck et al. 2017; Ringler et al. 2018). A tendency to use new pools whenever they are discovered could gradually produce a spatial pattern where the parent frogs travel farther and farther away from their home areas, which might additionally reduce future competition for mates and territories with their offspring. It is particularly surprising that the territorial males invest so much time in tadpole transport, because leaving the territory unattended may lead both to loss of mating opportunities and loss of the territory to competitors. In addition, movement may increase the predation risk (Paluh et al. 2014; also see observed predation in Fig. S2). The fact that frogs nevertheless travel such long distances suggests that the benefits, which remain to be quantified, outweigh the potential costs.

The reproductive behavior of many tropical frogs has been shaped by a trade-off between larger pools with high predation pressure and small pools with low food availability (Magnusson and Hero 1991; Duellman and Trueb 1994; McKeon and Summers 2013). In response to these trade-offs, some poison frog species became specialists of very small pools and evolved extended parental care, such as egg-feeding (for a review see Summers and McKeon 2004; Brown et al. 2010; Summers and Tumulty 2013). These species appear to have restricted space-use and often hold territories that include tadpole deposition sites (Donnelly 1989; Pröhl and Hödl 1999; Poelman and Dicke 2007; Brown et al. 2009). Many other poison frogs use pools of variable size and do not provide extended parental care, but the factors shaping their behavior are poorly understood. Ancestral poison frogs probably lived close to streams and did not need to travel far for tadpole deposition (Weygoldt 1987; Summers and McKeon 2004). Therefore, we speculate that long-distance tadpole transport between multiple terrestrial pools is another derived form of parental care that has been in part shaped by the adaptive benefits of parent-assisted offspring dispersal.

Our results also support the idea that poison frogs have a good spatial knowledge of the surrounding area and probably remember the exact locations of the pools themselves, as demonstrated in previous studies (Pašukonis et al. 2014, 2016, 2018; Beck et al. 2017). The movement trajectories of tadpole carriers were directed to small pools sometimes several hundred meters away from home. Given the well-developed spatial memory and the observed movements patterns, it seems unlikely that frogs were not able to detect the nearby pools and accidentally encountered the distant ones. How frogs discover the small distant pools in the first place remains unknown, but exploration during non-reproductive phases and olfactory cues might play a role (Pašukonis et al. 2016; Beck et al. 2017). We further hypothesize that the highly developed navigational abilities and spatial memory have coevolved with the long-distance shuttling of tadpoles.

Parental mobility may have a strong influence on offspring dispersal in a variety of animals outside poison frogs. Natural history observations suggest that in at least two rainforest frogs from Papua New Guinea (Bickford 2002) and one cave breeding Jamaican frog (Diesel et al. 1995), the transport of fully developed froglets serves a specific dispersal function. Transport of young is also common in arachnids and mammals, while many birds move together with their offspring. It has been long acknowledged that parents influence offspring dispersal decisions through conflict and competition (Motro 1983; Starrfelt and Kokko 2010), but more studies on individual movement patterns might reveal that parental spatial behavior also plays a significant role in offspring dispersal.

## Electronic supplementary material

Below is the link to the electronic supplementary material.

Supplementary material 1 (MP4 25056 kb)

Supplementary material 2 (DOCX 2140 kb)

Supplementary material 3 (CSV 3 kb)

Supplementary material 4 (XLSX 24 kb)

Supplementary material 5 (XLSX 20 kb)
