# Supplementary material for: How far do tadpoles travel in the rainforest? Parent-assisted dispersal in poison frogs
Source: Evol Ecol. 2019 Jul 5;33(4):613–23. doi: 10.1007/s10682-019-09994-z (PMC6647546; doi:10.1007/s10682-019-09994-z)
Supplement: Supplementary file 2 — Supplementary material 2 (DOCX 2140 kb) [file 10682_2019_9994_MOESM2_ESM.docx]

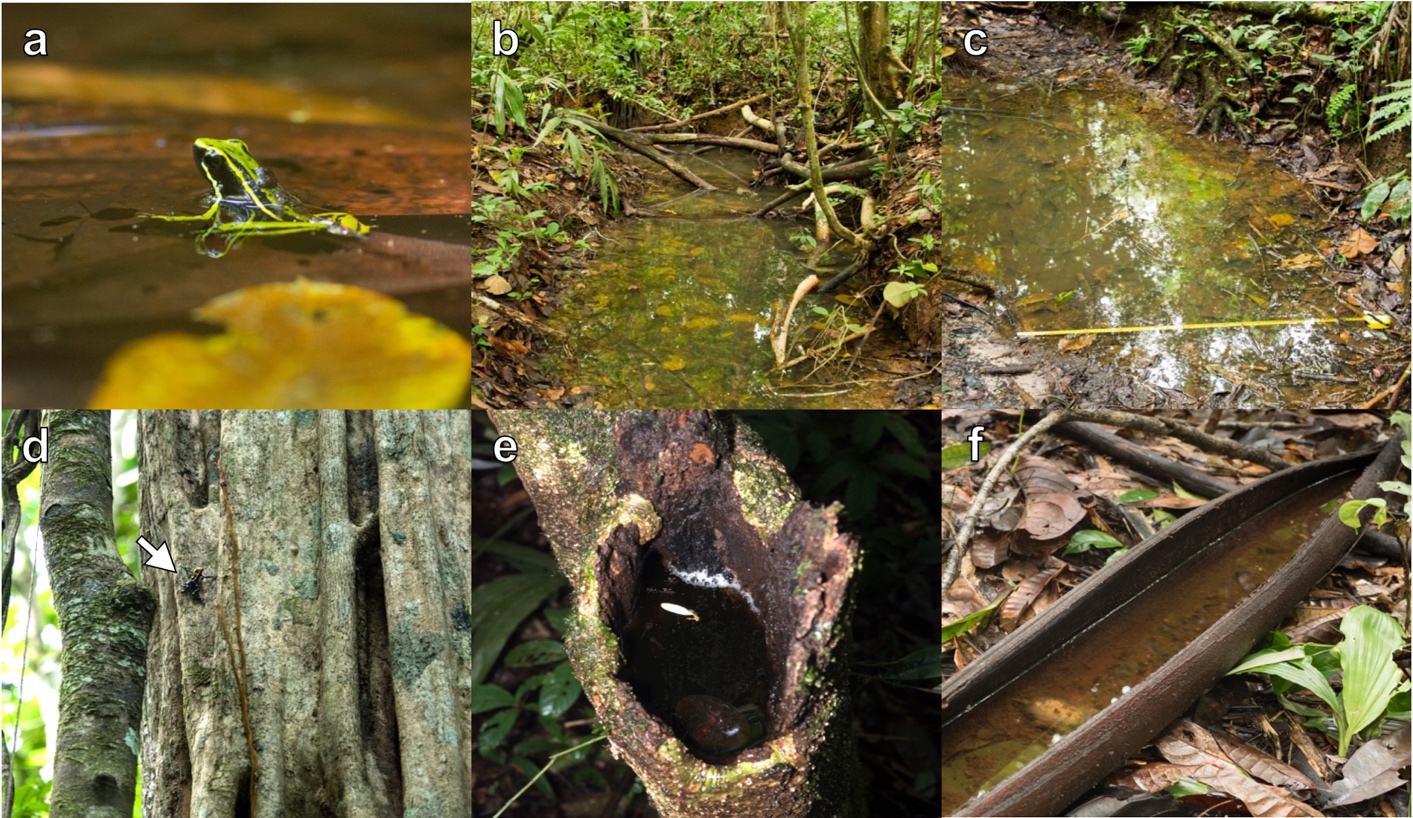


Supplementary figure S1. Photographs illustrating the aquatic habitat used for tadpole deposition by **a**, **b**, **c** *A. trivittata* and by **d**, **e**, **f** *D. tinctorius.* **a** Tracked male *A. trivittata* depositing tadpoles in a standing water pool inside **b** a stream bed. **c** A pool of standing water used by *A. trivittata*. Yellow measuring tape indicates one meter. **d** Tracked male *D. tinctorius* (indicated by white arrow) climbing a tree seven meters above ground to reach a water-filled tree hole. Examples of small **e** arboreal and **f** terrestrial pools used by *D. tinctorius* males.


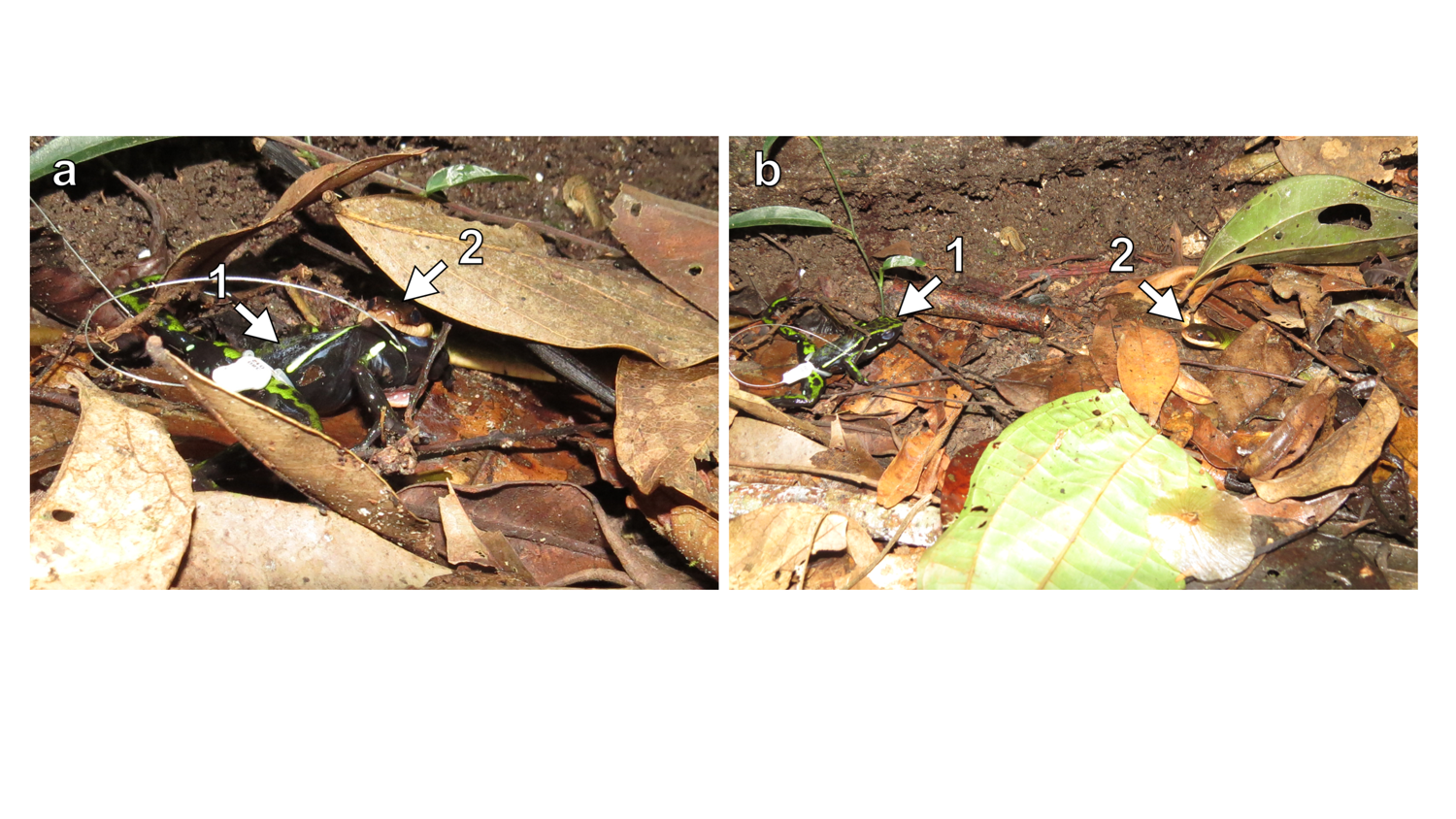


Supplementary figure S2. Two photographs showing the predation of male *A. trivittata* wearing a radio-transmitter (white arrow 1) by a colubrid snake *Erythrolamprus reginae* (white arrow 2). **a** Immobilized frog was discovered in the jaws of a snake soon after tadpole deposition several meters from the stream. **b** The snake released the dead frog after disturbance by the observers.


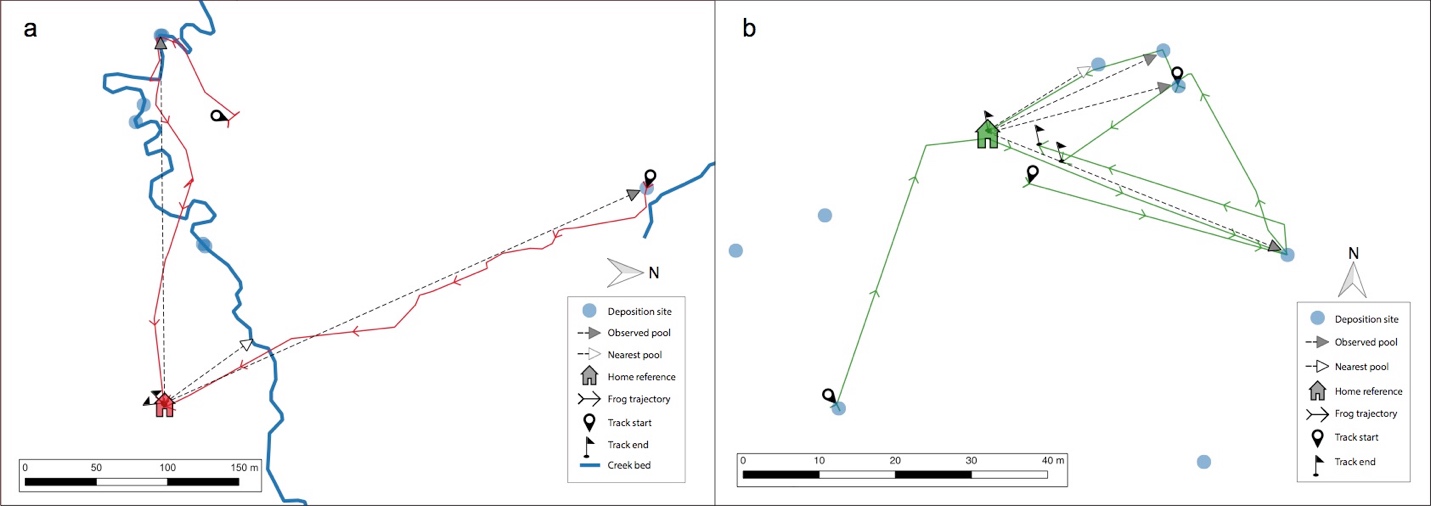
Supplementary figure S3. Example maps showing the movements during tadpole transport and the distances measured from the home reference to observed and nearest pools for two individuals: **a** one *A. trivittata* male during two tadpole transport events and **b** one *D. tinctorius* male during three tadpole transport events. Blue circles represent confirmed tadpole deposition sites; dashed lines with grey arrowheads represent observed pool distance; white arrowheads represent nearest pool distance; house symbols represent approximated start location of the tadpole transport; each line corresponds to the frog trajectory during each transport event; pin symbols correspond to the beginning and flags to the end of each transport event; blue line marks the creek bed, which provided continuous deposition sites for *A. trivittata*. The shown trajectories do not represent complete movement patterns because frogs were first detected already outside their home areas and near the deposition sites. Note the difference in map scales.
